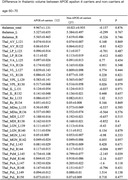# The relationship between *apoe* ε4 and cognitive function and pulvinar medial volume in patients age between 60 to 70 Years

**DOI:** 10.1002/alz.089171

**Published:** 2025-01-03

**Authors:** Jiajing Wu, Jiayu Wang, Linlin Li, Lei Chen, Qiansen Feng, Feiyan Wang, Ziqi Wang

**Affiliations:** ^1^ Nursing School of Zunyi Medical University, Zunyi China; ^2^ The Clinical Hospital of Chengdu Brain Science Institute, MOE Key Lab for Neuroinformation, School of Life Science and Technology, University of Electronic Science and Technology of China, Chengdu China; ^3^ The Fourth People’s Hospital of Chengdu, Chengdu China; ^4^ The Fourth People’s Hospital of Pengzhou, Chengdu China

## Abstract

**Background:**

To study the morphological characteristics of the thalamus in cognitively normal people with apolipoprotein E epsilon 4 (APOE ε4), and to explore whether it is affected by neuropsychiatric symptoms in patients aged between 60 and 70 years, and to provide evidence for the early brain structural changes in Alzheimer’s disease.

**Method:**

Clinical assessment, neuropsychological assessment, blood text and MRI examination were performed in 101 cognitively normal elderly patients in a tertiary psychiatric memory clinic in Chengdu. The cognitive function and thalamic volume of APOE ε4 carriers (n = 30) and APOE ε4 non‐carriers (n = 71) were compared using an independent sample T‐test. Multiple linear regression was used to analyze the association between thalamic and neuropsychiatric symptoms in APOE ε4 carriers.

**Result:**

The left pulvinar medial and right pulvinar medial volumes were significantly lower in 60 to70 year old APOE ε4 carriers than in non‐carriers (left pulvinar medial P = 0.039, right pulvinar medial P = 0.037). Long delayed recall score was not associated with left pulvinar medial or right pulvinar medial volume in individuals with APOE ε4 carriers (P > 0.05).

**Conclusion:**

The morphological changes of the pulvinar medial structure were associated with APOE epsilon 4 carriers at 60 to 70 years of age. There was no association between neuropsychiatric symptoms and pulvinar medial volume in APOE ε4 carriers.